# Post-transcriptional Control of Tumor Cell Autonomous Metastatic Potential by CCR4-NOT Deadenylase CNOT7

**DOI:** 10.1371/journal.pgen.1005820

**Published:** 2016-01-25

**Authors:** Farhoud Faraji, Ying Hu, Howard H. Yang, Maxwell P. Lee, G. Sebastian Winkler, Markus Hafner, Kent W. Hunter

**Affiliations:** 1 Metastasis Susceptibility Section, Laboratory of Cancer Biology and Genetics, National Cancer Institute, National Institutes of Health, Bethesda, Maryland, United States of America; 2 School of Medicine, Saint Louis University, Saint Louis, Missouri, United States of America; 3 Center for Biomedical Informatics and Information Technology, Center for Cancer Research, National Cancer Institute, National Institutes of Health, Bethesda, Maryland, United States of America; 4 School of Pharmacy, University of Nottingham, Nottingham, United Kingdom; 5 Laboratory of Muscle Stem Cells and Gene Regulation, National Institute of Arthritis and Musculoskeletal and Skin Diseases, National Institutes of Health, Bethesda, Maryland, United States of America; Cancer Research Center of Lyon, FRANCE

## Abstract

Accumulating evidence supports the role of an aberrant transcriptome as a driver of metastatic potential. Deadenylation is a general regulatory node for post-transcriptional control by microRNAs and other determinants of RNA stability. Previously, we demonstrated that the CCR4-NOT scaffold component *Cnot2* is an inherited metastasis susceptibility gene. In this study, using orthotopic metastasis assays and genetically engineered mouse models, we show that one of the enzymatic subunits of the CCR4-NOT complex, *Cnot7*, is also a metastasis modifying gene. We demonstrate that higher expression of *Cnot7* drives tumor cell autonomous metastatic potential, which requires its deadenylase activity. Furthermore, metastasis promotion by CNOT7 is dependent on interaction with CNOT1 and TOB1. CNOT7 ribonucleoprotein-immunoprecipitation (RIP) and integrated transcriptome wide analyses reveal that CNOT7-regulated transcripts are enriched for a tripartite 3’UTR motif bound by RNA-binding proteins known to complex with CNOT7, TOB1, and CNOT1. Collectively, our data support a model of CNOT7, TOB1, CNOT1, and RNA-binding proteins collectively exerting post-transcriptional control on a metastasis suppressive transcriptional program to drive tumor cell metastasis.

## Author Summary

The majority of human cancer related death is due to the effects of metastasis, the process of cancer dissemination to and growth in distant organs. Primarily due to its complexity, the metastatic process remains incompletely understood. This complexity stems from the tumor cell’s dependence on multiple cellular and molecular systems for the successful colonization of distant sites. In this study, we demonstrate that one of the factors that contributes to metastatic progression is the control of tumor cell RNA stability. Previously, we demonstrated that a structural component of the CCR4-NOT transcription regulatory complex was an inherited metastasis susceptibility gene. Here we demonstrate that one of the enzymatic components of the CCR-NOT complex, *Cnot7*, is also a metastasis-associated gene, and that enzymatic activity of *Cnot7* is required for its promotion of metastatic disease. These results suggest that large-scale control of RNA abundance may be modulating specific metastasis-related transcriptional programs, and that inhibition of specific RNA deadenylases may be a viable avenue in the development of anti-metastatic therapeutics.

## Introduction

Metastasis is a complex process in which tumor cells disseminate from the primary tumor site to form life-threatening lesions at distant sites. To successfully complete the metastatic process tumor cells must activate a series of molecular functions. These include motility and invasion to escape the primary site and penetrate the parenchyma at the secondary organ, anti-apoptotic programs to survive anoikis during transit through the lymphatic or hematic vasculature, and proliferative programs to establish clinically relevant macroscopic lesions [1]. Each of these programs requires the action of multiple genes in a coordinated fashion. As a result, control of transcriptional programs in the metastatic cascade has been the focus of many studies over the past decade. For example, activation of embryonic programs through up-regulation of transcription factors is thought to be important in the migratory and invasive steps of the metastatic cascade [2, 3]. Post-transcriptional control of metastasis-associated genes by microRNAs has also been the subject of a variety of studies [e.g. [4, 5]]. Activation or suppression of these pleiotropic factors, through mutation, amplification, or deletion, therefore plays critical roles in tumor evolution and progression.

In addition to activation or suppression of whole transcriptional programs, factors that significantly alter transcriptional units might also alter the ability of a tumor cell to complete one or more of the steps of the metastatic cascade. Studies in recent years have demonstrated an important role for inherited polymorphism in gene expression programs [6, 7] suggesting that inherited factors can significantly influence tumor phenotypes. Our laboratory previously demonstrated that inherited polymorphisms significantly influence metastatic outcome [8] and that inheritance plays a role in the establishment of transcriptional profiles that discriminate patient outcome [9]. More recently we have integrated gene expression analysis and susceptibility genetics studies to identify co-expressed transcriptional networks associated with metastatic disease. One such network was centered on *Cnot2*, a scaffolding component of the CCR4-NOT RNA deadenylase complex [10]. *In vivo* validation studies demonstrated that varying CNOT2 levels significantly influenced tumor metastatic capacity and implicated the CCR4-NOT complex as a novel determinant of tumor cell metastatic potential [10].

The CCR4-NOT complex is a modular, multifunctional protein complex highly conserved in eukarya [11]. Components of CCR4-NOT are found both in the nucleus and cytoplasm, and mediate transcriptional and post-transcriptional regulatory functions [12, 13]. In mammalian cells, CCR4-NOT has reported roles in epigenetically mediated transcriptional regulation [14], nuclear hormone receptor-mediated transcription [15], and initiation of transcript decay by deadenylation [16–18]. These observations suggest that the CCR4-NOT complex is a pleiotropic regulator of transcript abundance.

*Cnot2* depletion has been shown to disrupt CCR4-NOT deadenylase activity [19] which may be expected to alter metastasis-associated transcriptional programs. The absence of CNOT2 catalytic activity led us to hypothesize that other CCR4-NOT effector functions may drive metastasis. Moreover, the co-expression network analyses that identified *Cnot2* also implicated the CCR4-NOT deadenylase *Cnot8* and its binding partner *Tob1* as candidate metastasis driving genes [10], suggesting that CCR4-NOT deadenylase function may be an important determinant for metastatic progression.

Deadenylation, the progressive 3’-to-5’ shortening of the polyA tail, is a rate-limiting step of transcript destabilization [20]. Translational inhibition and transcript decay mediated by microRNAs [21], AU-rich elements [22], RNA binding proteins [23–25], and nonsense-mediated decay [26] occur through the recruitment of deadenylase complexes. The CCR4-NOT complex therefore plays an important role in maintaining mRNA equilibrium and coordinated control of transcriptional programs. We previously demonstrated that modulation of transcriptional elongation, mediated by *Brd4* [27], significantly altered the metastatic capacity of mammary tumor cells. In this study we extend these results by demonstrating that transcriptional decay, mediated by the deadenylation activity of *Cnot7* is also an important determinant in tumor progression. Our findings are consistent with the existence of post-transcriptional regulatory deadenylase complexes that promote metastasis by destabilizing metastasis suppressive transcriptional programs. Importantly, our work identifies CNOT7 deadenylase activity as a novel therapeutic target for anti-metastatic therapy.

## Results

### *Cnot7* depletion suppresses tumor cell autonomous metastatic capacity

**Fig 1:**
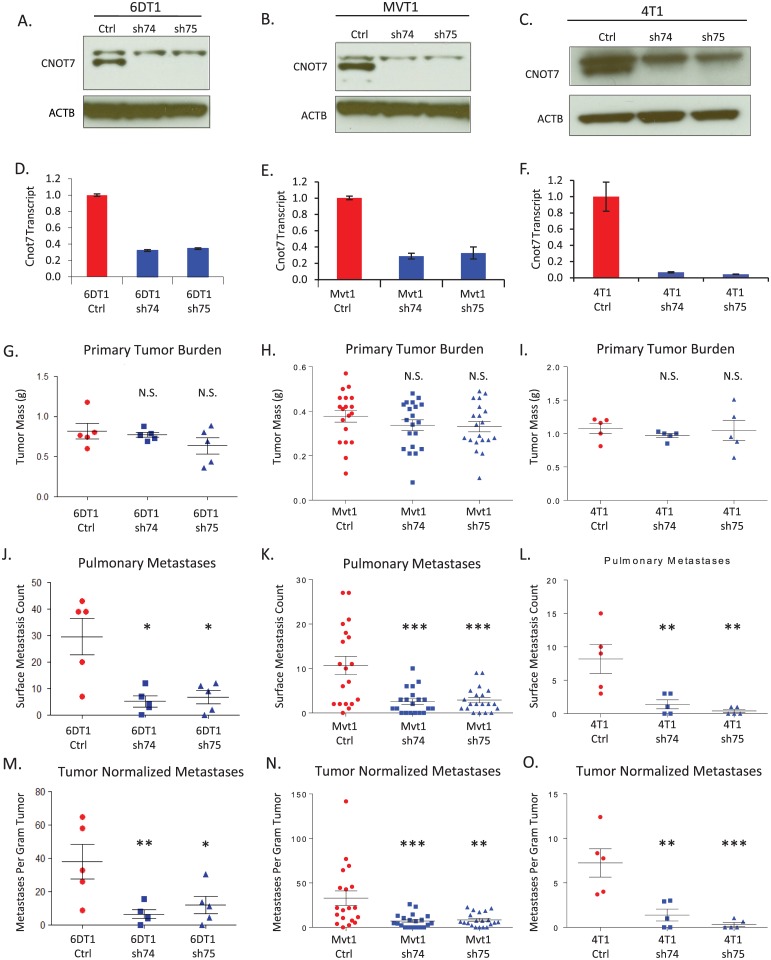
Results of *Cnot7* knockdown experiments for the cell lines 6DT1, Mvt1, and 4T1. Characterization of shRNA knockdowns at the protein (A-C) and mRNA (D-F) levels are shown across the top of the figure. The upper band in the western blots is a non-specific band that appears with some batches of α-CNOT7 antibody. The mass of orthotopically implanted tumors at necropsy is displayed in panels G, H, and I. The number of macroscopic pulmonary surface metastases at necropsy for each cell line is presented in panels J, K, and L. The number of macroscopic pulmonary surface metastases after normalization for primary tumor burden is displayed in panels M, N, and O. N.S. = not significantly different. * = p< 0.05; ** = p<0.01; *** = p<0.001.

Metastasis assays by orthotopic implantation were performed to test if perturbation of *Cnot7* or *Cnot8* expression alters metastatic capacity. *Cnot7* was knocked down in three independent murine mammary tumor cell lines [28, 29] by stable transduction of short hairpin RNAs (shRNAs), resulting in reductions of protein and transcript abundance (Fig 1). *Cnot8* was knocked down with the same method in 6DT1 and Mvt1 cells. After selection, transduced cells were implanted into the fourth mammary fat pad of syngeneic mice. At assay endpoint (t = 30 days), *Cnot8* knockdown did not produce consistent results between the tested cell lines (S1 Fig) and therefore was not included in further studies. *Cnot7* knockdown consistently reduced pulmonary metastasis without significant effect on primary tumor mass *in vivo* (Fig 1).

**Fig 2:**
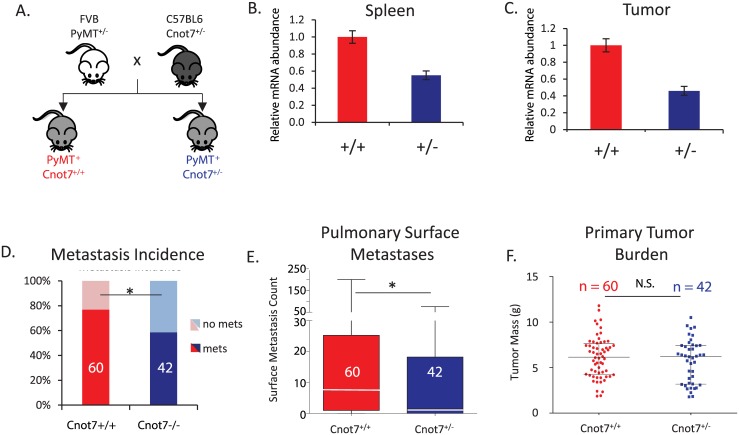
(A) Design of the MMTV-PyMT x *Cnot7* knockout mouse cross experiment. (B) Relative expression of *Cnot7* mRNA in the spleens of control and heterozygous knockout animals. (C) Relative expression of *Cnot7* mRNA in bulk primary tumors of control and heterozygous knockout animals. (D) Incidence of macroscopic pulmonary metastases (mets) for the PyMT^+^ *Cnot7*^*+/+*^ and PyMT^+^ *Cnot7*^*+/-*^ animals. The number of animals in each group is indicated by the numbers in the histogram bars. (E) Mean number and standard deviation of metastases observed for the PyMT^+^ *Cnot7*^*+/+*^ and PyMT^+^ *Cnot7*^*+/-*^ animals. Median values for each genotype are indicated by white line across the histogram bar. (F) Total tumor mass for the PyMT^+^ *Cnot7*^*+/+*^ and PyMT^+^ *Cnot7*^*+/-*^ animals. N.S. = not significantly different. * = p< 0.05.

To validate these results in an independent experimental system we next assessed the effect of diminished *Cnot7* expression in an autochthonous transgene-driven metastasis model. The MMTV-PyMT transgenic mammary tumor model [30] was bred to *Cnot7* hemizygous knockout [31] mice to produce PyMT^+^ *Cnot7*^+/-^ and PyMT^+^ *Cnot7* wild type mice (Fig 2A). Quantitative real time polymerase chain reaction analysis confirmed that *Cnot7*^*+/-*^ mice expressed *Cnot7* transcript approximately two-fold lower than wildtype mice in the spleen and tumor (Fig 2B & 2C). Consistent with observations in the orthotopic metastasis model, deletion of one copy of *Cnot7* significantly reduced the metastatic incidence and burden with no effect on primary tumor mass (Fig 2D–2F).

**Fig 3:**
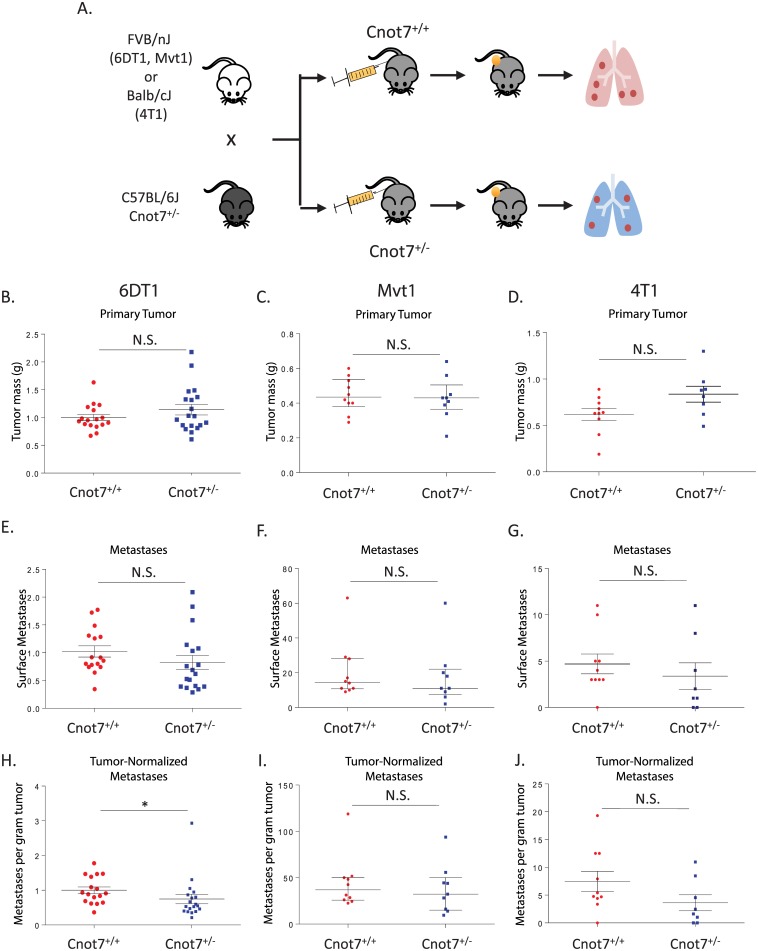
Design of the orthotopic implantation assays to test for stromal effects of *Cnot7* knockdown (A). Dot plots showing the primary tumor mass of each animal are shown for the cell lines 6TD1 (B), Mvt1 (C) and 4T1 (D). Surface pulmonary metastasis counts for each cell line are displayed in panels E, F, and G. Metastasis counts after normalization by primary tumor burden are displayed in panels H, I, and J. N.S. = not significantly different. * = p< 0.05.

To test if reduction of *Cnot7* expression in the stroma influenced metastasis, we then crossed C57BL/6J *Cnot7*^+/-^ to FVB/NJ or Balb/cJ mice to generate *Cnot7*^+/-^ and *Cnot7*^+/+^ mice that are immune-tolerant to the FVB- or BALB-derived tumor cells, respectively (Fig 3A). Wild type tumor cells were then orthotopically implanted into *Cnot7*^+/-^ and *Cnot7*^+/+^ mice and animals were aged for 28 days prior to necropsy. No consistent difference in tumor mass or metastasis was observed (Fig 3B–3J) suggesting the primary role of *Cnot7* in metastatic progression is tumor cell-autonomous.

**Fig 4:**
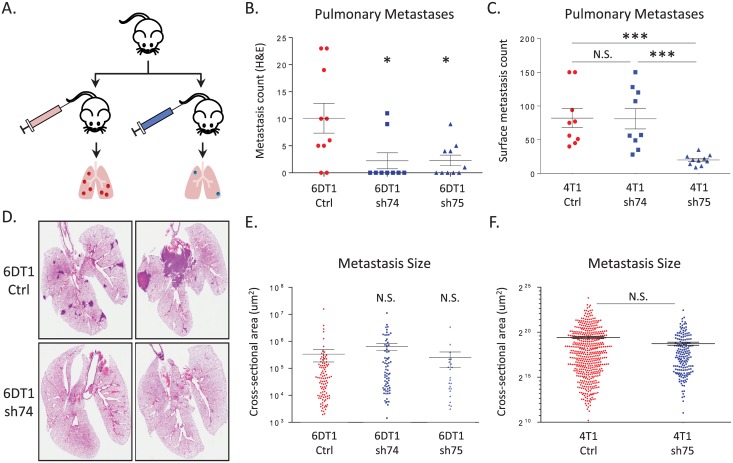
(A) Schematic of the experimental metastasis assay. Surface pulmonary metastases counts for 6DT1 are displayed in (B) and in (C) for 4T1. D) Representative H&E stained lung sections are shown for the 6DT1 experiment. Relative metastasis size is indicated in panel (E) for 6DT1 and (F) for 4T1. N.S. = not significantly different. * = p< 0.05; *** = p< 0.001.

Tumor cell colonization of distant organs is thought to be the rate-limiting step of the metastatic cascade [32, 33]. Lung colonization assays were therefore performed by intravenous injection of *Cnot7*-depleted tumor cells into the tail vein of mice (Fig 4A). Three of four *Cnot7* knockdown conditions resulted in significant suppression of lung colonization (Fig 4B–4D). Cross-sectional area of metastatic lesions was subsequently measured in hematoxylin and eosin (H&E) stained lung sections to determine if differences in colonization occurred secondary to proliferative differences. No difference in metastatic size between control and *Cnot7*-depleted cells was observed for either 6DT1 or 4T1 cells (Fig 4E & 4F). These results are consistent with a role of *Cnot7* promoting early stages of lung colonization in a proliferation-independent manner.

### *In vitro* analysis of the effect of *Cnot7* depletion

To investigate the potential cellular mechanisms underlying the suppression of metastatic capacity upon *Cnot7* depletion proliferation, motility, and colony formation in low attachment conditions were assessed. *Cnot7* depletion resulted in a consistent reduction of cellular proliferation in all three cell lines tested (S2 Fig) although, as noted above, this proliferative suppression was not observed in the *in vivo* orthotopic implantation assays.

Motility assays were performed on *Cnot7* depleted 6DT1 and Mvt1 cells. *Cnot7* knockdown in Mvt1 cells resulted in a reduced motility in a wound healing assay. In contrast, *Cnot7* knockdown in 6DT1 using the same shRNA constructs showed no consistent difference in motility as measured by wound healing assay (S2 Fig). Similar discrepancies across cell lines were observed in soft agar assays, where *Cnot7* depletion in 6DT1 resulted in significant reduction of colony formation while no difference was observed in Mvt1 cells (S2 Fig). Due to the lack of consistency among the *in vitro* assays, wound healing and soft agar assays were not performed for 4T1 *Cnot7* depleted cell lines. Overall the ambiguous results of the *in vitro* assays suggested that further investigations into the mechanisms of the role of *Cnot7* in metastatic disease would be best examined *in vivo*. Further efforts therefore focused on metastatic capacity based on orthotopic transplant assays.

### *Cnot7*-mediated metastasis promotion is dependent on its deadenylase activity

**Fig 5:**
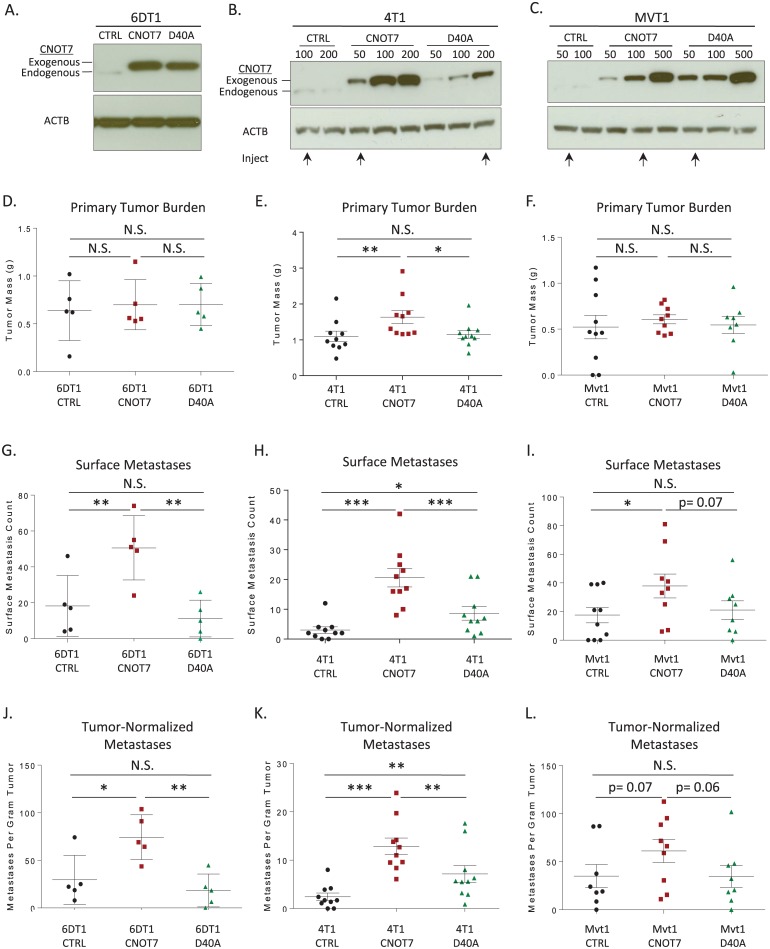
Western blots showing the relative expression of exogenously expressed CNOT7 and the D40A mutant in 6DT1 (A), 4T1 (B) and Mvt1 (C). The ectopically expressed CNOT7 migrates slightly higher than the endogenous band due to the addition of the FLAG epitope tag. The cell lines injected for 4T1 and Mvt1 experiments are indicated below the panels by arrows. Primary tumor burden for the CNOT7 or D40A mutant cell lines are indicated in panels D-F. Surface pulmonary metastases (G-I) and metastases normalized for primary tumor mass (J-L) are displayed on the last two rows of the figure. N.S. = not significantly different. * = p< 0.05; ** = p< 0.01; *** = p< 0.001.

The CCR4-NOT complex has been implicated in multiple cellular functions, including RNA deadenylation and degradation as well as transcriptional control [12]. To determine whether *CNOT7*-mediated metastasis promotion was deadenylase-dependent, we stably expressed the wild type and a deadenylase-inactive point mutant (D40A) [34–36] in mammary tumor cell lines. Transduced cultures were selected for approximately equal expression of CNOT7 and D40A protein (Fig 5A–5C), and used for *in vivo* orthotopic transplant assays.

Ectopic expression of CNOT7 or the D40A mutant showed no differences in primary tumor mass in Mvt1 or 6DT1 cells (Fig 5D & 5F). However, CNOT7 but not D40A significantly promoted metastatic burden for 6DT1 and Mvt1 (Fig 5G, 5I, 5J and 5L). In 4T1 cells, a statistically significant increase in tumor mass was observed for *CNOT7* but not for D40A expressing cells (Fig 5E). Normalization of metastatic burden by tumor mass to account for difference in primary tumor growth still resulted in a significant difference in metastatic capacity for *CNOT7* expressing 6DT1 or 4T1 cells and borderline significance in Mvt1 cells. Furthermore, although the 4T1 D40A expressing cells exhibited increased metastasis compared to control, a significant reduction of metastatic capacity was observed compared to *CNOT7*-wild type expressing cells (Fig 5H and 5K). Overall these results are consistent with a major role of the deadenylase function of *CNOT7* in modulating metastatic capacity of mammary tumor cell lines.

### CNOT7 modulation of metastasis requires interaction with the CCR4-NOT-associated adaptor proteins TOB1 and CNOT1

**Fig 6:**
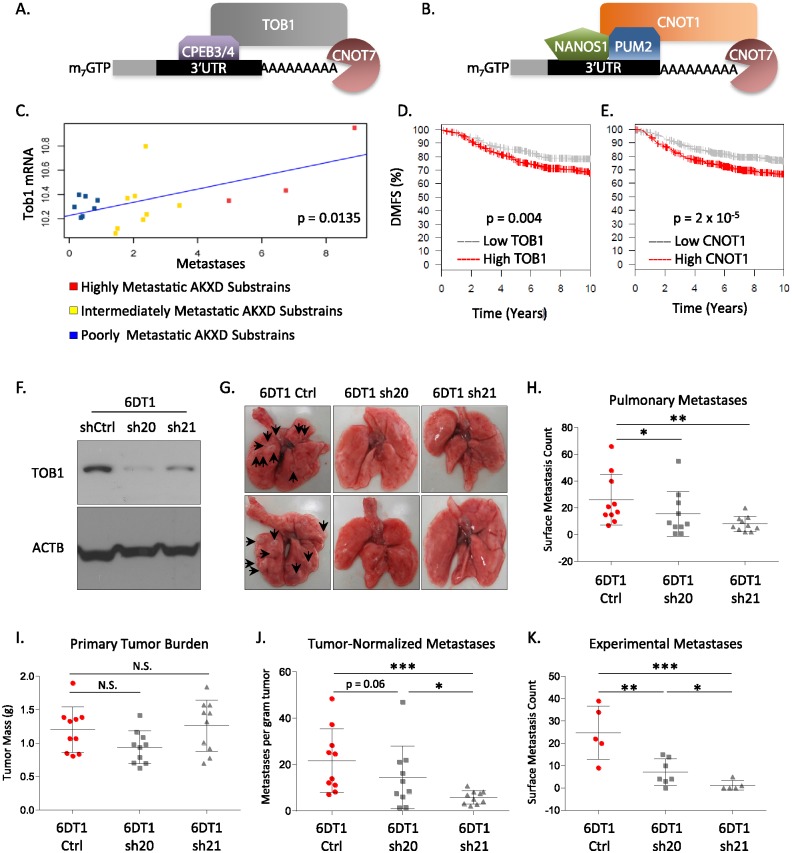
Schematic models of mRNA recruitment to CNOT7 by TOB1 (A) or CNOT1 (B). Correlation plot of *Tob1* expression levels with metastatic capacity in the AKXD recombinant inbred mouse panel (C). Kaplan-Meier analysis of the expression of *TOB1* (D) or *CNOT1* (E) and distant metastasis free survival (DMFS) in the GOBO meta-dataset. Western blot demonstrating the reduction of TOB1 mRNA levels by shRNA knockdown (F). Representative whole mounts of lungs from the *Tob1* knockdown spontaneous metastasis assay (G). Dot plots for surface pulmonary metastases from the *Tob1* knockdown spontaneous metastasis assay (H). Primary tumor burden for the orthotopically implanted tumors in the *Tob1* knockdown assay (I). Surface pulmonary metastases for the *Tob1* knockdowns after normalization by primary tumor weight (J). Dot plots of pulmonary metastases for the experimental tail vein injection assay (K). N.S. = not significantly different. * = p< 0.05; ** = p< 0.01; *** = p< 0.001.

CNOT7 is a non-specific RNA-binding deadenylase protein [35]. Specificity for transcripts is mediated by sequence specific RNA binding proteins that interact with the CCR4-NOT complex. TOB1 is an adaptor protein that recruits CNOT7 to specific RNA-binding proteins, while CNOT1 is a scaffolding protein for the CCR4-NOT complex [37, 38] (Fig 6A and 6B). Previous work from our laboratory found that *Tob1* expression was correlated with metastasis in the [PyMT x AKXDn]F1 mice [10] (Fig 6C). In addition, human breast cancer datasets–available through the Gene expression-based Outcome for Breast cancer Online (GOBO) database, an expression array-based meta-analysis data set of 1,881 breast cancer patients [39]–also showed that high expression of either *TOB1* or *CNOT1* correlated with poor survival (Fig 6D & 6E). Furthermore TOB1 has previously been associated with poor distant metastasis free survival in breast cancer patients [40].

Orthotopic metastasis assays were conducted to test the role of the CCR4-NOT adaptor proteins in metastatic disease. Attempts to generate stable *Cnot1* knockdown cells were unsuccessful and therefore orthotopic assays were not performed. *Tob1* knockdown in 6DT1 cells (Fig 6F) showed diminished metastasis with no effect on primary tumor mass (Fig 6G–6J). Experimental metastasis assays showed that *Tob1* knockdown suppressed lung colonization of 6DT1 cells (Fig 6K), consistent with *Tob1* acting at the same stage of the invasion-metastasis cascade as *Cnot7*. In 4T1 cells, *Tob1* knockdown suppressed tumor mass and pulmonary metastasis (S3 Fig). Significant suppression of metastasis was observed after normalizing by tumor mass, consistent with an effect other than just on tumor growth (S3 Fig).

**Fig 7:**
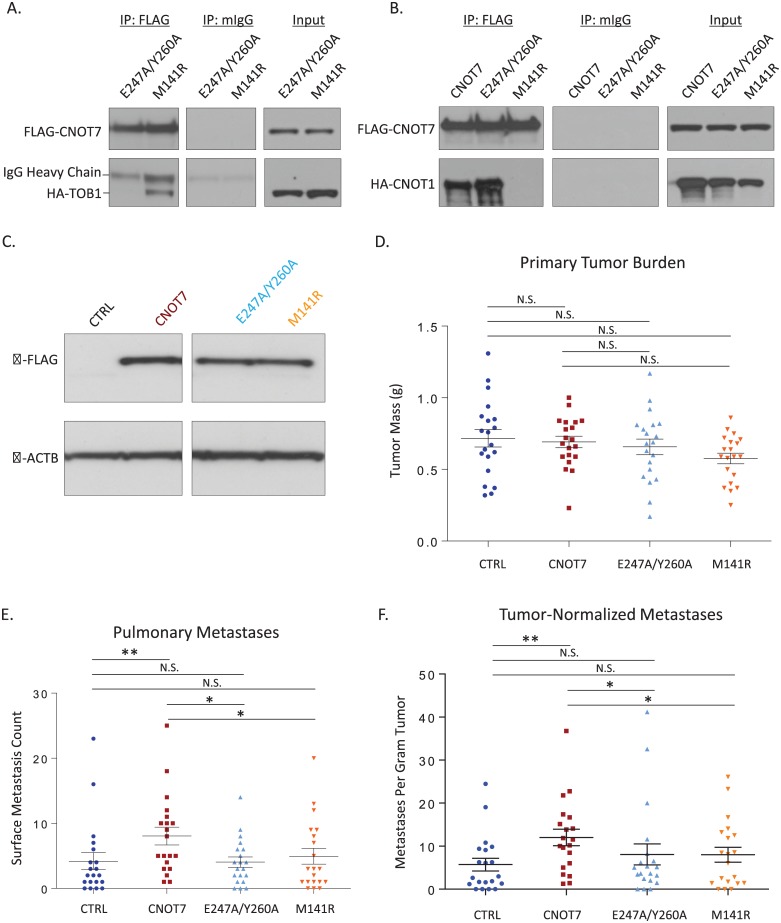
Co-immunoprecipitation of the *Cnot7* mutants confirming the specific disruption of interaction with TOB1 by the E247A/Y260A mutant (A). Co-immunoprecipitation of the *Cnot7* mutants confirming the specific disruption of interaction with CNOT1 by the M141R mutant (B). Western blot confirming the equal expression of *Cnot7* constructs in cells used for spontaneous metastasis assays (C). Dot plot of the primary tumor mass for *Cnot7* wildtype and mutant cells orthotopically implanted into mice (D). Surface metastasis plot results of orthotopically implanted 4T1 cells expressing mutant *Cnot7* constructs (E). Dot plot of the surface pulmonary metastasis count for orthotopically constructs normalized for primary tumor burden (F).

To test if CNOT7-mediated metastasis promotion was dependent on a complex with TOB1 or CNOT1, we constructed expression vectors of *CNOT7* mutants that disrupted interaction with TOB1 or CNOT1. CNOT7 E247A/Y260A mutations have previously been shown to disrupt interaction with BTG/TOB family proteins but maintain interaction to CNOT1 [36]. Conversely, CNOT7 M141R substitution abolishes complex formation between CNOT7 and CNOT1 [34], but interaction with TOB1 has not previously been assessed. Co-immunoprecipitation (IP) experiments were therefore performed to address this question. As expected, IP of CNOT7 E247A/Y260A but CNOT7 M141R co-precipitated CNOT1. In contrast, the CNOT7 M141R mutant retained the ability to co-precipitate TOB1 but no longer interacted with CNOT1 (Fig 7A & 7B), indicating that the two mutant constructs specifically disrupted interaction with the TOB1 or CNOT1 adaptor proteins.

*CNOT7* and the mutant constructs were then expressed in 4T1 cells to achieve equal levels of CNOT7, CNOT7 E247A/Y260A, and CNOT7 M141R protein (Fig 7C) and the cells were then implanted orthotopically into mice. No significant changes in the rate of tumor growth or primary tumor mass at endpoint were observed (Fig 7D). Consistent with previous observations, overexpression of CNOT7 promoted tumor cell metastatic potential but expression of CNOT7 E247A/Y260A or CNOT7 M141R showed no change in metastasis compared to control (Fig 7E and 7F). These results indicate that CNOT7-mediated metastasis promotion depends on contact with both TOB1 and CNOT1.

### *Cnot7* inversely-regulated transcripts are enriched for specific RNA binding motifs

The above results suggest that *Cnot7* mediates its effect on metastasis by modulating the RNA equilibrium. Therefore, to gain a better idea of the global gene expression program affected by *Cnot7*, we identified mRNAs that exhibited an inverse relationship with *Cnot7* expression in 4T1 cells in which *Cnot7* was knocked down or over-expressed. Array-based transcriptome analysis yielded 842 significantly dysregulated transcripts (p<0.01, S1 Table). Of these, 514 transcripts were upregulated upon *Cnot7* knockdown and down-regulated upon *CNOT7* overexpression (t<0, S1 Table; *Cnot7*-anticorrelated transcripts). 3’ untranslated regions (3’UTRs) were then interrogated for known consensus RNA-binding protein (RBP) sequence motifs enriched in the *Cnot7* inversely-correlated transcripts (S4 Fig). The inversely correlated transcripts showed enrichment for the cytoplasmic polyadenylation element (CPE) [41], Pumilio binding element (PUM) [42], Nanos response elements (NRE) [43], and cleavage and polyadenylation stimulation factor binding element (CPSF) [44]. In contrast neither permissive (AUUUA) nor stringent (UUAUUUAUU) AU-rich elements (ARE) [45] were found to be enriched indicating that, in 4T1 cells, *Cnot7* preferentially mediates the degradation of a specific subset of mRNAs (S4 Fig).

### RIP-Seq identifies a prognostic set of mRNAs directly regulated by *Cnot7*

**Fig 8:**
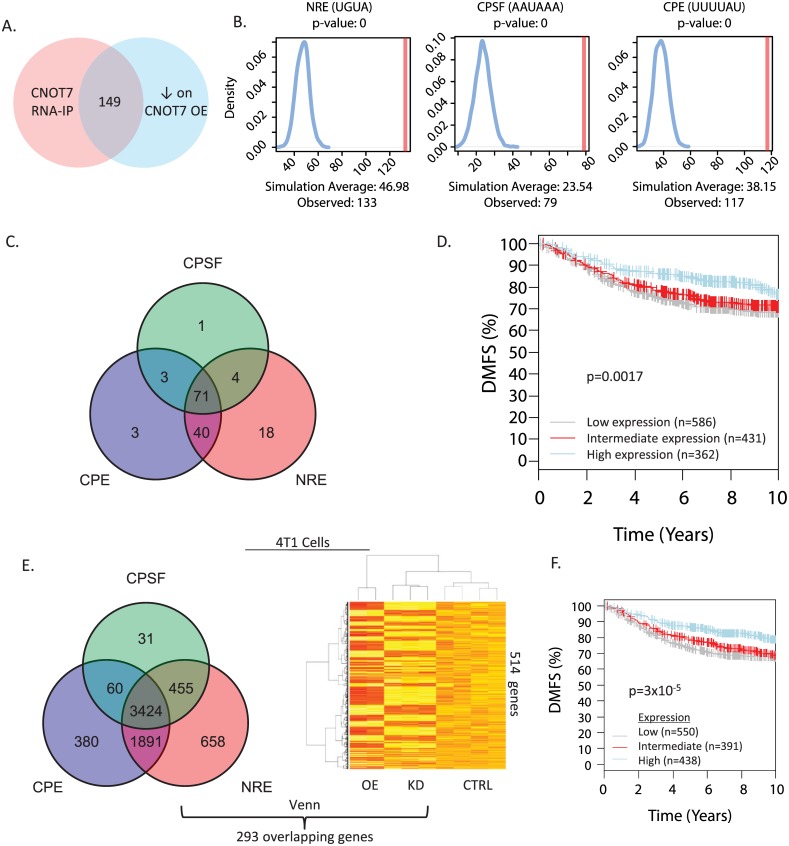
Overlap between transcripts identified by RNA immunoprecipitation (RNA-IP) and the microarray analyses (A). RNA binding protein motif enrichment for the RIP-seq transcripts (B). Overlap analysis of the CPE, CPSF and NRE RNA binding protein motifs in the 149 transcripts in common between the RIP-seq and microarray experiment (C). Kaplan-Meier analysis of the GOBO meta-dataset using as a weighted gene signature the 46 represented human orthologs of the 71 tripartite motif-possessing transcripts detected by RIP-seq (D). Results of genome-wide analysis of transcripts containing predicted CPE, CPSF and NRE RNA protein binding motifs (E). Overlap of this gene set with the *Cnot7* inversely correlated genes from the microarray analysis identified 293 genes in common. Kaplan-Meier analysis of GOBO meta-dataset using the 293 CPE/CPSF/NRE tripartite transcripts as a weighted gene signature (F).

To identify transcripts directly regulated by CNOT7, RNA-immunoprecipitation was performed using the anti-FLAG (M2) antibody in 4T1 cells overexpressing FLAG-CNOT7. Co-precipitated RNA was subjected to high throughput sequencing (RIP-seq). 149 transcripts showed enrichment relative to input and control (M2 RIP in 4T1 empty vector cells not expressing FLAG-CNOT7) and showed an inverse correlation with *Cnot7* expression (Fig 8A). These transcripts showed 3’UTR enrichment for CPE, CPSF, and NRE sites (Fig 8B). PUM and ARE sites were not considered since fewer than 10 of the 149 genes contained these binding motifs. Seventy-one of 149 transcripts (48%) possessed CPE, CPSF, and NRE sites (Fig 8C, S2 Table) suggesting that CPEB, CPSF, and Nanos family proteins may collectively constitute specificity factors that cooperatively drive metastasis by targeting CNOT7 to metastasis-associated transcripts.

We next tested if the 71 transcripts that shared the tripartite motif were prognostic in human breast cancer data sets. Forty-six (65%) human orthologs of the CPE/CPSF/NRE containing genes were present in GOBO [39] (S3 Table). These 46 genes were applied as a gene signature, weighted by their inverse correlation with CNOT7 expression, to determine whether they could discriminate patient outcome. Consistent with the possibility that CNOT7 drives progression by degrading metastasis suppressing mRNAs, high expression of the signature was correlated with favorable distant metastasis free survival (DMFS, Fig 8D).

### Global screen for putative CPE/CPSF/NRE *Cnot7* target genes

Since CNOT7-bound and inversely regulated transcripts were reproducibly enriched for the CPE/CPSF/NRE motifs, we speculated that this tripartite motif may specify CNOT7 target transcripts, which are enriched for metastasis-associated genes. We thus interrogated the entire genome for transcripts that possessed CPE, CPSF, and NRE 3’UTR elements, and filtered this set for those transcripts expressed in 4T1 cells. This analysis identified 3424 genes (12.5% of the mouse genome, Fig 8E). Filtering this set for those inversely-correlated with *Cnot7* expression yielded 293 genes (S4 Table). Human orthologs for 217 (74%) of the 293 genes were available in GOBO, and were able to significantly discriminate DMFS (Fig 8F). High expression of this signature predicted favorable survival, suggesting that this set of tripartite motif containing, *Cnot7*-anticorrelated transcripts constituted a metastasis suppressive post-transcriptional program.

Subjecting this 293-gene set to Ingenuity Pathway Analysis identified cancer as the most highly represented disease annotation, with 247 (84%) transcripts previously annotated as cancer-associated. The top five represented canonical pathways associated with extravasation signaling, and cancer-associated HER2 signaling, HGF signaling, and MAPK-signaling. The top five upstream regulators in this network included *Kras*, *Erbb2*, and *Tgfb1* signaling (S5 Table). Gene sets regulated by each of these upstream regulators predicted distant metastasis free survival (S5 Fig). Consistent with our model, *Erbb2* and *Kras* are previously reported upstream signaling components that regulate the activity of CNOT7 adaptor and deadenylation cofactor TOB1, transducer of ERBB2 [46, 47].

## Discussion

CCR4-NOT is a highly conserved protein complex that has been implicated in diverse functions associated with gene regulation [12, 13]. It exists in both cytoplasmic and nuclear forms and is thought to play different roles depending on its subcellular localization and subunit composition [12]. In the nucleus the CCR4-NOT complex has been implicated in numerous activities, including chromatin modification, transcriptional elongation, RNA export, nuclear RNA surveillance and transcription-coupled DNA repair [14, 15]. In the cytoplasm the CCR4-NOT complex is thought to be the main RNA deadenylase, initiating both mRNA decay and translational repression by polyA tail shortening [12, 13]. The CCR4-NOT complex also participates in miRNA-mediated gene silencing through interactions with the GW182/Argonaute complex [21]. This large, multi-functional protein complex therefore has the potential to play a variety of important roles in establishing and maintaining cellular function and response to extracellular cues.

Previously our laboratory implicated the CCR4-NOT complex as an important determinant for metastatic mammary cancer. Generation of co-expressed gene maps from mouse strains with differing inherited sensitivity for pulmonary metastasis identified a network module centered on the CCR4-NOT component *Cnot2* that was capable of discriminating breast cancer patient outcome. Furthermore, *in vivo* modeling demonstrated that suppression or over-expression of CNOT2 within tumor cells resulted in enhanced or reduced pulmonary metastases, respectively, indicating that *Cnot2* has metastasis suppressing activities [10]. CNOT2 however does not have known enzymatic activities [48]. CNOT2 coordinates the interaction of CNOT3 with the core CCR4-NOT complex, as well as additional regulatory molecules including HDAC3 [14]. Despite lacking catalytic function, CNOT2 has been shown to be an important positive regulator of CCR4-NOT deadenylase activity [19], cellular apoptosis, and mouse embryonic stem cell pluripotency [49]. The contribution of *Cnot2* to metastatic capacity through the CCR4-NOT complex could therefore occur through a variety of CCR4-NOT molecular functions.

In this study we have begun to dissect the role of CCR4-NOT in mammary tumor metastasis by investigating the role of RNA deadenylation in tumor progression. The integrated genetics and gene expression analysis that initially identified *Cnot2* also implicated other genes associated with RNA deadenylation (*Cnot8*, *Angel2*, *Tob1*) as potential modulators of metastatic disease, suggesting that this function of CCR4-NOT might be a critical determinant [10]. Bioinformatics analysis indicated that *CNOT8* and *TOB1* were associated with distant metastasis free survival in human patients. We therefore selected *Cnot8* and its highly conserved paralog *Cnot7* to determine whether the deadenylation function of CCR4-NOT plays a critical role in tumor progression.

*Cnot7* and *Cnot8* are members of the DEDD superfamily of deadenylases [35]. Both genes are expressed ubiquitously in tissues of adult animals [50] and are thought to have overlapping functions [36]. Biochemical studies suggest that only one of the two proteins exist in the CCR4-NOT complex at a time, suggesting unique functions for the mutually exclusive CNOT7- or CNOT8-containing complexes, in addition to redundant functions [34, 51]. This interpretation is consistent with the differing results observed for the shRNA knockdowns of the two genes in our studies. *Cnot7* knockdown had little or no effect on primary tumor growth, indicating that its role in tumor progression is related to the metastatic process. In contrast *Cnot8* suppressed both primary tumor growth and metastatic disease, suggesting a more general role in regulating tumor cell proliferation. Further elucidation of the commonalities and differences in molecular pathways controlled by these two deadenylases would likely provide interesting insights into tumor growth and progression.

Importantly, due to the multifunctional nature of the CCR4-NOT complex, the ability of *Cnot7* to promote metastatic disease was dependent on deadenylase activity. Point mutations eliminating enzymatic activity or that disrupted interactions mediating recruitment of RNA binding proteins to the CCR4-NOT complex suppressed the pro-metastatic activity of CNOT7. Knockdown of the adaptor protein TOB1, which acts as a bridge between CNOT7 and RNA binding proteins CPEB3 and CPEB4, which recruit RNAs to the complex for deadenylation, had similar effects. Attempts to knockdown CNOT1, which is responsible for recruitment of the NANOS1 and PUM2 RNA binding proteins, was unsuccessful. However, due to the central role of CNOT1 in the CCR4-NOT complex and increased apoptosis in CNOT1-depleted cells [52–54], this result was not unexpected. Taken together however, the point mutant and *Tob1* depletion results suggest that the majority of the effect on metastasis was likely due to the influence of CCR4-NOT on RNA equilibrium or translational efficiency, rather than on the other many functions ascribed to the complex.

The CCR4-NOT complex is thought to be one of two general deadenylase complexes in mammalian cells [18]. However, the difference in phenotypes for *Cnot7* and *Cnot8* knockdowns suggest that the different complexes likely target overlapping subsets of RNAs within the cell [36]. Alternatively, the two paralogs may be differentially expressed and regulate different post-transcriptional programs in different cell types. To gain a better understanding of what subset *Cnot7*-containing CCR4-NOT complexes target global gene expression analysis was performed in two independent experiments. We focused specifically on genes that were inversely correlated with *Cnot7* levels to enrich for those that were likely direct targets rather than those dysregulated due to secondary effects on the transcriptome. Since transcript recruitment to the CCR4-NOT complex is specified by RNA binding proteins a screen for RNA binding proteins was performed [23–25, 38, 55][56]. This screen revealed an enrichment of some but not all of the known RNA binding protein motifs, suggesting that the *Cnot7* metastatic suppressive program is mediated by specific RNA binding partners. Further investigation of these RNA binding proteins and their RNA targets will likely provide additional insights into the molecular pathways important for metastatic progression.

Encouragingly enrichment of three of the four RNA binding protein motifs (CPE, CPSF and NRE) was replicated in the *Cnot7* RIP-seq experiment, providing more direct evidence of the interaction of the CNOT7-containing CCR4-NOT complex with the *Cpsf*, *Nanos* and *Cpeb* families of RNA binding proteins. Furthermore, almost half (71/149) of the RNAs identified by the RNA-immunoprecipitation contained all three motifs. When used as a weighted gene signature, this set of transcripts was capable of discriminating metastasis outcome in human breast cancer. Global transcriptome analysis in 4T1 mammary tumor cells revealed only 293 expressed genes bearing all three RNA binding protein motifs, consistent with previous findings of only limited numbers of genes changing after *Cnot7* knockdown [36]. Like the 71 genes identified by RIP-Seq, the 293 triple motif containing genes effectively discriminate outcome in breast cancer patients, suggesting enrichment of genes and molecular functions associated with breast cancer patients. This interpretation was further supported by pathway analysis, which identified previously known metastasis-associated functions such as extravasation and *Tgfb1* signaling as enriched in the 293-gene set.

Overall this study supports the hypothesis that in addition to initiation of specific transcriptional programs, such as epithelial-to-mesenchymal transition, degradation of specific RNAs may play an important role in the establishment of metastatic capacity. Further investigations of the RNA binding proteins that recruit transcripts for deadenylation and studies into possible roles of the other CCR4-NOT deadenylase subunits *Cnot6* and *Cnot6l* in metastatic progression may reveal additional important insights into tumor autonomous metastatic mechanisms. Moreover, these results also suggest that targeting *Cnot7* deadenylase activity may be useful for anti-metastatic therapy. *Cnot7* knockout animals are viable, with limited known phenotypes, indicating that pharmaceutical suppression of *Cnot7* deadenylase activity may not be unacceptably toxic in the clinic. If true, this would provide a novel class of therapeutic agents to suppress colonization or the emergence of disseminated but dormant tumor cells, ultimately leading to a reduction in the morbidity and mortality associated with metastatic disease.

## Materials and Methods

### Ethics statement

The research described in this study was performed under the Animal Study Protocol LCBG-004, approved by the NCI Bethesda Animal Use and Care Committee. Animal euthanasia was performed by cervical dislocation after anesthesia by Avertin.

### Cell lines and culture conditions

The mouse mammary carcinoma cell lines 4T1, 6DT1, Mvt-1 [28] (provided by Dr. Lalage Wakefield) and human embryonic kidney HEK293 cells were cultured in Dulbecco’s Modified Eagle Medium (DMEM) (Gibco) supplemented with L-Glutamate (Gibco), 9% fetal bovine serum (FBS) (Gemini BioProducts), and 1% Penicillin and Streptomycin (P/S) (Gemini BioProducts).

### Lentiviral transduction

Two milliliter suspensions of 10^5^ cells were incubated at 37°C in 5% CO_2_ overnight. Cells were then infected with lentivirus suspension, and selected 30 hours post-infection with 5mg/mL blasticidin for over-expression (Invitrogen) constructs or 10ug/mL (shRNA) puromycin for shRNA constructs.

### Co-immunoprecipitation

Co-immunoprecipitation was conducted as described in [57] using mouse origin anti-FLAG and Protein G Dynabeads magnetic beads (Invitrogen).

### *In vitro* cell line assays

Proliferation and wound healing assays were performed on the Incucyte ZOOM (Essen BioScience) system following the previously described protocols [58]. For soft agar assays, 5,000 trypsinized cells were seeded in triplicate in 0.4% low-melting-point agarose (Sigma) on top of a 1% agarose layer and colonies enumerated 21 days later.

### Expression array

Array-based transcriptome profiling of *Cnot7*-knockdown and CNOT7-overexpressing 4T1 tumor cells was performed on Affymetrix GeneChip Mouse Gene 1.0 ST arrays by the Microarray Core in the NCI Laboratory of Molecular Technology.

### Library preparation and RNA sequencing

Library preparation was performed using the NEBNext Ultra Directional RNA Library Prep Kit for Illumina with NEBNext multiplexing oligos using manufacturer’s protocol. RNA sequencing was conducted on the Illumina HiSeq 2500.

### RNA isolation, reverse transcription and Real-Time Polymerase Chain Reaction

RNA was isolated from tumors and cell lines using RNeasy kit (Qiagen) or TriPure (Roche) and reverse transcribed using iScript (Bio-Rad). Real-Time PCR was conducted using VeriQuest SYBR Green qPCR Master Mix (Affymetrix).

### Lentivector cloning

cDNA sequences of human FLAG-CNOT7, FLAG-CNOT7-D40A, and FLAG-CNOT7-E247A/Y260A were describe previously [36]. Lentiviral expression vectors were produced with Multisite Gateway recombination. An entry clone using the murine Pol2 promoter was recombined with the cDNA entry clone and N-terminal entry clone encoding the MYC (EQKLISEEDL) or FLAG (DYKDDDDK) epitope tag into a Gateway destination vector pDest-658. pDest-658 is a modified version of the pFUGW lentiviral vector which contains the enhanced polypurine tract (PPT) and woodchuck regulatory element (WRE) to provide higher titer virus. It also contains an antibiotic resistance gene for blasticidin resistance. Entry clones were subcloned by Gateway Multisite LR recombination using the manufacturer’s protocols (Invitrogen). Expression clones were transformed into E. coli STBL3 cells to minimize unwanted LTR repeat recombination, and verified by agarose gel electrophoresis and restriction digest. Transfection-ready DNA for the final clones was prepared using the GenElute XP Maxiprep kit (Sigma). A control vector (8166-M24-658) was generated by standard Gateway LR recombination of a stuffer fragment made up of a non-coding DNA into the pLenti6-V5-DEST vector (Invitrogen). CNOT7 and CNOT7-D40A lentivector constructs were generated by the Protein Expression Laboratory and the Viral Technology Group in NCI, Frederick, MD.

Site directed mutagenesis was employed to generate CNOT7-M141R mutant using the primers listed in S6 Table. The cDNA segment containing these mutations was subcloned into the wild type *CNOT7* lentivector by restriction digest and ligation.

### Native RNA immunoprecipitation

4T1 cells expressing 8166-M24-658 control vector or FLAG-CNOT7 constructs were grown to ~85% confluence in ten 15cm culture plates. Cells were then rinsed with 15mL ice cold sterile PBS, scraped off, pelleted (~1mL pellet), and snap frozen in liquid nitrogen. Cells were thawed on ice and lysed in 3.5mL lysis buffer (100mM NaCl, 5mM MgCl_2_, 10mM HEPES (pH 7.3), 0.5% NP40, 200 units RNasin (Promega), Protease inhibitor cocktail (Roche)). The resulting 4mL of cell lysate was spun down twice at 21,000*g for 20 minutes at 4°C. 40uL of lysate was saved 1% input to confirm immunoprecipitation. 200uL of lysate was saved for 5% RNA-seq input and was purified by TriPure (Roche) RNA extraction. 25uL beads per 1mL lysate of Protein G Dynabeads were blocked with 1mL 0.5% BSA in PBS at room temperature for 20 minutes then washed twice with 1mL NT2 wash buffer (50mM Tris-HCl (pH 7.4), 150mM NaCl, 1mM MgCl_2_, 0.05% NP40). 24ug antibody was added to each sample and incubated at 4°C overnight then beads were added to each sample and rotated for 30 minutes at room temperature. Beads were washed four times in 1mL NT2 buffer. Eighty five percent of beads were subjected to RNA extraction with TriPure. Protein from the remaining 15% of beads was eluted with Laemli buffer to confirm immunoprecipitation.

### Immunoblot and antibodies

Protein was extracted with Pierce lysis buffer, vigorously homogenized, and incubated on ice for twenty minutes. 20ug lysate per sample in NuPage LDS Sample Buffer and NuPage Reducing Agent (Invitrogen) was used for western blotting. PVDF membrane (Millipore) containing transferred proteins was incubated overnight in solution of 5% milk protein, tris-buffered saline supplemented with 0.05% Tween-20, and primary antibody. The membrane was then incubated with horse-radish peroxidase linked anti-mouse (GE Healthcare), anti-rat, or anti-rabbit (Santa Cruz Biotechnology) IgG secondary antibodies. Immunoblot was visualized using Amersham ECL Prime Western Blotting Detection System and Amersham Hyperfilm ECL (GE Healthcare). Rabbit origin anti-CNOT7 was a generous provided by G. Sebastiaan Winkler. Commercial antibodies used in this study include rabbit origin anti-TOB1 (GeneTex), rabbit origin anti-CNOT1 (Protein Tech), rat origin anti-HA (Roche), mouse origin monoclonal anti-FLAG (M2, Sigma).

### Animal studies

Female FVB/NJ or Balb/cJ mice from Jackson Laboratories were injected at 6–8 weeks of age. Two days prior to orthotopic injections, cells were placed in non-selective media. On the day of injection, 1x10^5^ cells were injected orthotopically into the fourth mammary fat pad of age-matched virgin females. After 30 days the mice were euthanized by intraperitoneal injection of 1mL Tribromoethanol with subsequent cervical dislocation. Primary tumors were resected, weighed, and snap frozen in liquid nitrogen. Lungs were resected, surface metastases were counted; lungs were inflated with 10% nitrate-buffered formalin and sent for sectioning and staining. For tail vein injection, 10^5^ were injected into the lateral tail vein, mice were euthanized 22 days post-injection. All procedures were performed under the Animal Safety Proposal (LCBG-004) and approved by the NCI-Bethesda Animal Care and Use Committee.

### Statistical analysis

Statistical analysis comparing two samples were conducted using the Mann-Whitney test on Prism Version 5.03 (GraphPad Software, La Jolla, CA). Multiple-comparison data was analyzed by Kruskal-Wallis test with post-hoc Conover-Inman correction for multiple analyses by R-script. Survival data was conducted with the Mantel-Cox test on Prism.

RBP motif sites were mapped on the 3’UTR regions of genome genes (27305 mRNA FASTA format) by using a Perl script. RBP motif site enrichment analysis was performed by the random sampling the genes from genome in the same number (the final overlapping gene number) and calculate the numbers of the gene with the RBP motif site and of the RBP motif site and repeat the sampling 1000 times. The p-value was estimated by one tail t-test. Differential gene expression analyses were done by t-test using the software package R.

### Data access

Array-based gene expression and RIP-seq studies from this study have been submitted to the NCBI Gene Expression Omnibus (GEO; http://www.ncbi.nlm.nih.gov/geo/ under the accession numbers GSE73296 (array) and GSE73366 (RIP-seq).

## Supporting Information

S1 Fig Results of *Cnot8* knockdown *in vivo* studies. Relative *Cnot8* mRNA depletion by shRNA (arbitrary units)for 6DT1 and Mvt1 cells is shown in panels (A) and (B). Effects of shRNA knockdown are shown for the primary tumor for 6DT1 (C) and Mvt1 (D) are shown at the top of the figure. The macroscopic pulmonary surface metastases counts are shown in panels (E) and (F). Macroscopic pulmonary surface metastases after normalization for primary tumor mass are shown in panels (G) and (H). N.S. = not significantly different. * = p< 0.05; ** = p<0.01; *** = p<0.001.
(EPS)

S2 Fig Effect of *Cnot7* depletion on the proliferation of 6DT1, Mvt1 and 4T1 cells (A). Results of *Cnot7* knock down on migration of 6DT1 and Mvt1 cells, as measured by wound healing assays (B). Soft agar colony formation assays for 6DT1 and Mvt1 *Cnot7* knock down cell lines (C).
(EPS)

S3 Fig Relative knockdown of *Tob1* mRNA in 4T1 cells (A). Primary tumor burden after orthotopic transplantation of 4T1 *Tob1-*knockdown cells into BALB/c animals (B). Pulmonary surface metastasis count of 4T1 *Tob1-*knockdown tumors (C). Tumor normalized metastasis counts of 4T1 *Tob1-*knockdown cells (D).
(EPS)

S4 Fig Permutation strategy to identify over-represented RNA binding protein motifs in *Cnot7* inversely-correlated transcripts from microarray analysis (A). Results of motif search (B). The vertical red line represents the observed number of times a particular motif was found in the *Cnot7* inversely correlated gene set. The blue curve represents the predicted range based on 1000 permutations.
(EPS)

S5 Fig Kaplan-Meier analysis of the GOBO meta-dataset using pathway enriched subsets of the 293 CPE/CPSF/NRE tripartite 4T1 expressed transcripts. Gray lines indicate patients with tumors that expressed the individual gene signatures below the median value. Red lines indicate patients with tumors expressing the gene signatures above the median.
(EPS)

S1 Table Genes dysregulated by overexpression or knockdown of *Cnot7*. (XLS)

S2 Table Genes with negative expression correlations with Cnot7 whose mRNAs are associated with the CNOT7 protein. (XLS)

S3 Table Human-mouse gene symbol matching for GOBO weighted gene signature prognosis analysis. (XLSX)

S4 Table Genes with negatively correlated with Cnot7 expression that contain the CPE/CPSF/NRE tripartite RNA binding protein motif. (XLSX)

S5 Table Ingenuity Pathway Analysis of CPE/CPSF/NRE bearing, Cnot7 negatively correlated genes. (XLS)

S6 Table Primers and primer sequences used in this analysis. (XLS)
